# Alterations in miRNA Levels in the Dentate Gyrus in Epileptic Rats

**DOI:** 10.1371/journal.pone.0076051

**Published:** 2013-10-11

**Authors:** Anna Maria Bot, Konrad Józef Dębski, Katarzyna Lukasiuk

**Affiliations:** Nencki Institute of Experimental Biology, Polish Academy of Sciences, Warsaw, Poland; University of Modena and Reggio Emilia, Italy

## Abstract

The aim of this study was to characterize changes in miRNA expression in the epileptic dentate gyrus. Status epilepticus evoked by amygdala stimulation was used to induce epilepsy in rats. The dentate gyri were isolated at 7 d, 14 d, 30 d and 90 d after stimulation (n=5). Sham-operated time-matched controls were prepared for each time point (n=5). The miRNA expression was evaluated using Exiqon microarrays. Additionally, mRNA from the same animals was profiled using Affymetrix microarrays. We detected miRNA expression signatures that differentiate between control and epileptic animals. Significant changes in miRNA expression between stimulated and sham operated animals were observed at 7 and 30 d following stimulation. Moreover, we found that there are ensembles of miRNAs that change expression levels over time. Analysis of the mRNA expression from the same animals revealed that the expression of several mRNAs that are potential targets for miRNA with altered expression level is regulated in the expected direction. The functional characterization of miRNAs and their potential mRNA targets indicate that miRNA can participate in several molecular events that occur in epileptic tissue, including immune response and neuronal plasticity. This is the first report on changes in the expression of miRNA and the potential functional impact of these changes in the dentate gyrus of epileptic animals. Complex changes in the expression of miRNAs suggest an important role for miRNA in the molecular mechanisms of epilepsy.

## Introduction

Epilepsy is one of the most prevalent serious neurological disorders, affecting approximately 1% of the world’s population [1]. It is estimated that approximately 50 million people worldwide and 6 million in Europe alone have active epilepsy. Approximately 30% of these patients are drug refractory [2]. The health and indirect social costs associated with epilepsy are a considerable burden for society.

In many cases, epilepsy develops as the result of a brain damaging insult that initiates cascades of events, called epileptogenesis (latency period), after which recurrent spontaneous seizures occur [3]. Epileptogenic insult causes a number of changes that lead to the remodeling of neuronal circuits and subsequent seizures, however it is still not clear which of them are causative [4,5]. One method to elucidate the processes underlying epilepsy that has been successfully used is the analysis of global changes in the transcriptome to distinguish metabolic pathways affected by epileptic processes [6-10]. Very recently, general mechanisms of the regulation of gene expression, in addition to the gene expression patterns, have become of interest. In particular the role of epigenetic mechanisms including DNA methylation and miRNA, have been investigated [11,12].

miRNAs are small (20-24 bp), non-coding RNAs that regulate the expression of target genes by binding to the 3’-untranslated regions (UTR) of target mRNAs. It is estimated that miRNAs regulate as much as 60% of mRNAs and that one miRNA can target a few to several hundred genes [13], which makes miRNAs very powerful players in the regulation and fine-tuning of gene expression. Alterations in miRNAs levels can have pronounced effects.

Brain has an exceptionally high expression of miRNAs. Approximately 60% of all miRNA species are present in the brain [14]. This suggests a particularly significant role for miRNAs in brain physiology, as well as the possibility of serious consequences in cases of miRNA dysfunction. In fact, changes in the expression levels of individual miRNAs have been observed in physiological conditions, including learning and memory formation, neuronal development and plasticity, as well as pathological conditions, such as psychiatric diseases, neurodegeneration, ischemia, Alzheimer’s disease, and epilepsy [12,15-19].

Currently, data on the role of miRNAs in epilepsy are limited. Dysfunction of miRNA processing and decrease in miRNA levels as well as alterations in expression of individual miRNAs were observed in temporal lobe epilepsy patients with hippocampal sclerosis [20-22]. Few studies report changes in miRNA expression in the hippocampus of epileptic rats in pilocarpine or lithium-pilocarpine models of epilepsy [23-25].

In the present work we decided to investigate changes in the expression levels of miRNAs in the dentate gyrus, a structure that has been studied for a long time in the context of epilepsy and epilepsy development. For instance, in the epileptic dentate gyrus, the occurrence of abnormal neuronal plasticity, abnormal neurogenesis, alterations in GABA-mediated inhibition and neurodegeneration limited to certain populations of neurons have been observed [26-28]. Here we describe miRNA expression patterns at different times following epileptogenic stimulus in the animal model of temporal lobe epilepsy. Additionally we propose potential mRNA targets by evaluating the mRNA expression levels in the same tissue samples. This is the first report on changes in the expression of miRNA and the potential functional impact of these changes in the dentate gyrus of epileptic animals.

## Materials and Methods

### Animal surgery and status epilepticus induction

Adult male Sprague-Dawley rats (Medical Research Centre, Warsaw, Poland) weighing 290–320 g were used in this study. Animals were housed in a controlled environment (22°C ± 1°C, lights on 07:00–19:00) with free access to food and water. Starting from the day of surgery, each animal was housed in a separate cage. All animal procedures were approved by the Ethical Committee on Animal Research of the Nencki Institute and conducted in accordance with the guidelines established by The European Council Directives 2010/63/EU.

The amygdala stimulation model of temporal lobe epilepsy was used in this study [29].

Status epilepticus (SE) was triggered by electrical stimulation of the amygdala as previously described by Guzik-Kornacka et al. [30] with some modifications. Briefly, surgery was performed under isoflurane anesthesia (2-2.5% in 100% O_2_) preceded by the injection of butorphanol (Butomidor, Richter Pharma AG, Wells, Austria; 0.5 mg/kg i.p.). A stimulating and recording bipolar wire electrode (Plastic One Inc., Roanoke, VA, # E363-3-2WT-SPC) was implanted into the left lateral nucleus of the amygdala 3.6 mm posterior and 5.0 mm lateral to bregma, 6.5 mm ventral to the surface of the brain [31]. A stainless steel screw electrode (Plastic One Inc., Roanoke, VA, #E363/20) was implanted contralaterally into the skull over the right frontal cortex (3.0 mm anterior and 2.0 mm lateral to bregma) as a surface EEG recording electrode. Two stainless steel screw electrodes were placed bilaterally over the cerebellum (10.0 mm posterior and 2.0 mm lateral to bregma) as ground and reference electrodes. The socket contacts of all electrodes were placed in a multi-channel electrode pedestal (Plastic One Inc., Roanoke, VA, #MS363) which was attached to the skull with dental acrylate (Duracryl Plus). After 2 weeks of recovery, animals were electrically stimulated via the intra-amygdala electrode to evoke status epilepticus. Stimulation consisted of a 100-ms train of 1-ms biphasic square-wave pulses (400 µA peak to peak) delivered at 60 Hz every 0.5 s for 30 min. If the animal did not enter status epilepticus, stimulation was continued for an additional 10 min. The SE was stopped 1.5-2 h after stimulation via an intraperitoneal injection of diazepam (20 mg/kg). If the first dose of diazepam did not suppress SE, the animal received subsequent doses of diazepam at 5 mg/kg. Time-matched control animals had electrodes implanted but did not receive electrical stimulation.

Rats sacrificed at 7 d, 14 d, and 30 d after SE were monitored with video-EEG (Comet EEG, Grass Technologies, West Warwick, RI) continuously from the moment of stimulation until the end of the experiment. Rats sacrificed 90 d after SE were monitored for 5 d after stimulation and then for 2 weeks before the end of experiment to determine the frequency of spontaneous seizures. Spontaneous seizures were identified from EEG recordings by browsing the EEG manually on the computer screen. An electrographic seizure was defined as a high frequency (>8 Hz), high amplitude (>2x baseline) discharge lasting for at least 5 s. Latency to the ﬁrst spontaneous seizure, number and frequency of seizures, and number of epileptic animals in each group were evaluated.

For tissue collection, the rats were anaesthetized with CO_2_ and decapitated with a guillotine. The brains were removed from the skull and the hippocampi were isolated on an ice cold plate and placed in RNAlater solution (Ambion, AM7024) then stored at -20°C until use.

### miRNA isolation and profiling

The left dentate gyrus region was dissected under a dissection microscope. The isolation of total RNA including miRNA, was performed using the miRNeasy Mini kit (QIAGEN, # 217004) according to the manufacturer’s instructions. The sample concentration and contamination with protein or organic compounds were determined using a NanoDrop 2000 spectrophotometer (Thermo, Fisher Scientific). All samples had Abs 260/280 >2.0 and 260/230 >1.4. The quality of the total RNA was further verified using the an Agilent 2100 Bioanalyzer.

The miRNA array profiling experiment was performed at Exiqon Services, Denmark using the miRCURY LNA^TM^ microRNA Array 7^th^ targeting all microRNAs for human, mouse or rat registered in the miRBASE version 19.0 at the Sanger Institute. The data are available at Gene Expression Omnibus under the accession number GSE49850 (http://www.ncbi.nlm.nih.gov/geo/query/acc.cgi?acc=GSE49851).

### Transcriptome profiling

GeneChip® Rat Gene 1.1 ST arrays (Affymetrix, Santa Clara, CA, # 901627) were used for mRNA profiling. The same mRNA samples were used for miRNA and mRNA profiling. One hundred nanograms of total mRNA was used for cDNA synthesis using an Ambion WT Expression Kit (Life Technologies, # 4411974). Hybridization, washing and scanning were conducted according to Affymetrix guidelines for the GeneAtlas^TM^ instrument. The data are available at Gene Expression Omnibus under the accession number GSE49849 (http://www.ncbi.nlm.nih.gov/geo/query/acc.cgi?acc=GSE49851).

### Data analysis

The preliminary analysis of miRNA microarrays identifying upregulated and downregulated miRNAs was performed by the Exiqon Company. All calculations were performed with the software R/Bioconductor [32,33] primarily with the limma package [34]. For the expression analysis, the calculated p-values were based on moderated t-statistics. Furthermore, the Benjamini and Hochberg multiple testing adjustment method was applied to the p-values (FDR – False Discovery Rate).

The correlation, cluster and principal component analyses were performed in the R environment (version 15.1) [33].

Principal component analysis (PCA) was performed on all 292 microarray probes with an intensity greater than the background value using the function prcomp (package stats). A PCA plot (splom) was generated with the lattice package [35].

For the heatmap clustering of miRNAs with significantly different expression (FDR < 0.05) between all epileptic and sham-operated control animals, miRNAs were ordered with the clustering complete-linkage method together with the Pearson correlation distance measure. The heatmap diagram was generated with the gplots package [36]

Pearson correlation test was used to analyze the correlations between miRNAs with significantly different expression (FDR < 0.01) between stimulated and sham-operated control animals. A correlation plot was generated with the corrplot package [37].

The fuzzy c-means algorithm implemented in the Mfuzz package [38] was used to perform clusterization on all 292 probes that had expressions higher than the background. The functional analysis of miRNA belonging to individual clusters was performed using the Ingenuity Pathways analysis software (Ingenuity Systems, Redwood City, CA).

Analysis of the Affymetrix Rat Gene 1.1 ST arrays was performed using R/Bioconductor [33]. The microarrays were normalized with the Robust Multi-array Average (RMA) algorithm (oligo package version 1.22.0) [39]. The intensity of the genomic probes below the median intensity of antigenomic probes (considered as a background value) were corrected to this median value. Probes with intensity equal to or lower than the background in more than five samples were removed from analysis. Only probes that corresponded to a single gene were selected for further analysis. A one-way ANOVA was used to establish genes that were differentially expressed between groups.

The miRNA targets were selected using the Ingenuity Pathways analysis software (Ingenuity Systems, Redwood City, CA) containing a manually curated collection of miRNA-target interactions. Only mRNAs with significantly changing expression levels (p<0.05) in comparison to the time-matched controls were included. Of those, only mRNAs changing expression level in the opposite direction to that of the miRNAs were selected for presentation.

DAVID (version 6.7) [40,41] was used for the functional annotation of potential gene targets. The Biological Process Gene Ontology (GO) was analyzed using the Functional Annotation Clustering tool with the default *Rattus norvegicus* background. The resulting Annotation Clusters were named in a way that best described their GO terms.

## Results

We evaluated the expression of miRNA in the dentate gyrus of epileptic animals sacrificed at 7, 14, 30 and 90 days following amygdala stimulation-induced SE and time-matched sham controls. All stimulated animals included in the study developed spontaneous seizures and were therefore diagnosed with epilepsy. Rats from the 7 d, 14 d and 30 d groups were monitored with a video-EEG system continuously from the moment of stimulation until the end of the experiment, while animals from 90 d group were only monitored for 2 wks preceding tissue collection. The median number of seizures during the monitoring period was as follows: 1 (range 0-4) in the 7 d group, 11 (range 1-17) in the 14 d group, 11 (range 5-24) in the 30 d group, and 8 (range 3-17) in the 90 d group. The number of seizures during the last one week of monitoring was as follows: 2 (range 1-12) in 14 d group, 6 (range 2-14) in the 30 d group, and 4 (range 0-14) in the 90 d group.

**Figure 1:**
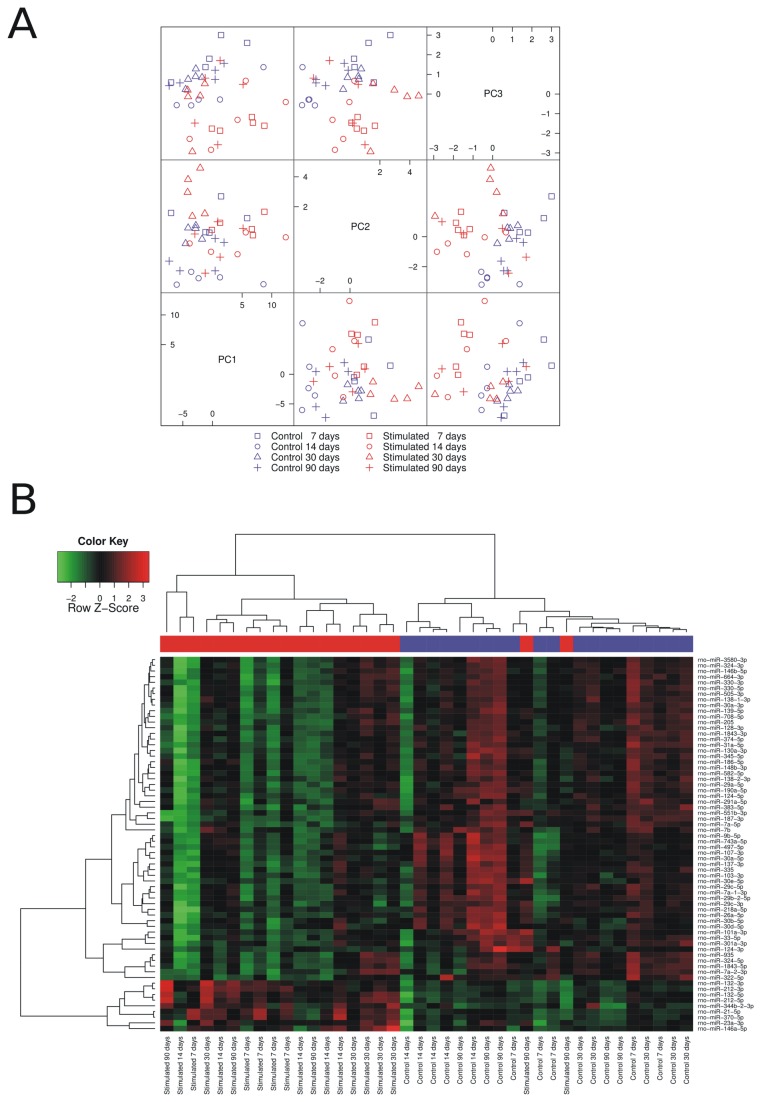
miRNA expression profiles in the dentate gyrus of epileptic and sham-operated control animals at different times after SE. (**A**) Principle Component Analysis (PCA) of microarray data derived from epileptic (red) and sham-operated control animals (blue) at 7 d (square), 14 d (circle), 30 d (triangle), and 90 d (cross) after status epilepticus. Each mark represents an individual animal. Note that epileptic animals are separate from the controls. (**B**) A heatmap of the 66 miRNAs with altered expression levels in epileptic animals (Table 1). Each column represents an individual animal and each row represents an individual miRNA. Colors on the heatmap represent the Z-score: higher – red, lower – green. Red color in the bar over the heatmap panel represents epileptic animals, and blue represents sham-operated control animals.

To detect similarities and differences between samples, PCA analysis was performed. The results of the PCA performed on all miRNA probes with an intensity greater than the background indicated, that the miRNA expression profiles of the sham-operated controls and stimulated animals were different (Figure 1A). The profiles of the epileptic animals were grouped together and are separate from the controls.

To detect prolonged changes in miRNA expression levels regardless of the time point, we analyzed the differences in the expression levels of individual miRNAs between all sham-operated controls and all stimulated animals. We detected significant changes in the expression of 66 miRNAs. Table 1 lists all miRNAs for which the expression levels differed between the sham-operated and epileptic animals at an adjusted p-value of 0.05. Nine miRNAs were upregulated, while 57 miRNAs were downregulated. The largest decreases in expression were observed in miR-187-3p, miR-551b-3p and miR-7a-5p at 0.61, 0.67 and 0.71 fold of the control levels respectively. The largest increases in expression occurred in miR-212-3p, miR-132-3p and miR-21-5p at 1.54, 1.52 and 1.41 fold of the control levels respectively (Table 1).

**Table 1:** List of miRNAs differentially expressed between all stimulated and all control animals (FDR<0.05).

**EXIQON probe**	**microRNA**	**logFC**	**FDR**
42627	miR-212-3p	0.62	0.0004
10937	miR-132-3p	0.6	0.003
147506	miR-21-5p	0.53	0.0028
145634	miR-132-5p	0.5	0.0062
148304	miR-212-5p	0.4	0.0062
10952	miR-146a-5p	0.35	0.0264
42744	miR-23a-3p	0.34	0.0243
148300	miR-370-5p	0.32	0.012
148099	miR-344b-2-3p	0.14	0.0394
11231	miR-345-5p	-0.15	0.0363
13150	miR-322-5p	-0.16	0.0062
42898	miR-124-5p	-0.16	0.0252
31388	miR-291a-5p	-0.16	0.0486
148614	miR-7a-2-3p	-0.16	0.0486
148278	miR-138-2-3p	-0.18	0.0429
42606	miR-330-3p	-0.2	0.0264
33902	miR-128-3p	-0.21	0.0429
14316	miR-664-3p	-0.21	0.0429
145825	miR-383-5p	-0.22	0.0221
42792	miR-29b-2-5p	-0.22	0.0243
11277	miR-7a-1-3p	-0.22	0.0243
46917	miR-205	-0.23	0.0243
148175	miR-1843-3p	-0.23	0.0264
42847	miR-497-5p	-0.23	0.0269
11041	miR-29c-3p	-0.23	0.0343
4390	miR-7b	-0.23	0.0377
168937	miR-138-1-3p	-0.23	0.0432
42669	miR-505-3p	-0.23	0.0432
19595	miR-30a-3p	-0.24	0.0252
148477	miR-1843-5p	-0.25	0.0178
148017	miR-743a-5p	-0.25	0.0377
18739	miR-186-5p	-0.25	0.0396
10919	miR-103-3p	-0.25	0.047
42477	miR-324-5p	-0.25	0.0482
145708	miR-324-3p	-0.27	0.0321
14328	miR-124-3p	-0.27	0.0363
145843	miR-330-5p	-0.28	0.0377
42800	miR-582-5p	-0.29	0.0199
10923	miR-107-3p	-0.29	0.0377
10306	miR-146b-5p	-0.29	0.0429
19585	miR-148b-3p	-0.3	0.0243
11065	miR-335	-0.3	0.0264
13143	miR-301a-3p	-0.3	0.0383
145638	miR-29a-5p	-0.3	0.0419
146112	miR-30b-5p	-0.3	0.0419
145742	miR-935	-0.3	0.0429
10138	miR-130a-3p	-0.31	0.0243
27536	miR-190a-5p	-0.32	0.0178
11052	miR-31a-5p	-0.33	0.002
148519	miR-3580-3p	-0.33	0.0264
14300	miR-29c-5p	-0.34	0.01
148131	miR-9b-5p	-0.35	0.0264
147198	miR-26a-5p	-0.39	0.0132
11018	miR-218a-5p	-0.4	0.008
145749	miR-137-3p	-0.4	0.0178
29190	miR-708-5p	-0.42	0.008
31026	miR-101a-3p	-0.42	0.047
146086	miR-30a-5p	-0.43	0.012
145859	miR-33-5p	-0.43	0.0176
19596	miR-30d-5p	-0.44	0.0178
145677	miR-139-5p	-0.47	0.0188
148098	miR-374-5p	-0.48	0.002
28191	miR-30e-5p	-0.48	0.0121
29490	miR-7a-5p	-0.49	0.0105
148644	miR-551b-3p	-0.57	0.0066
145637	miR-187-3p	-0.71	0.0001

miRNAs are ordered according to decreasing logFC

Heatmap of 66 miRNAs with altered expression levels in stimulated animals revealed different expression patterns between the control and epileptic animals (Figure 1B). Two main clusters generated by this analysis act as the first division branches, segregating the sham-operated control animals from the stimulated ones. The exceptions are two animals sacrificed at 90 d after stimulation, which were more similar to the sham-operated control animals. Within the cluster of stimulated animals, fractions of rats belonging to the 7 d group as well as those belonging to the 30 d group formed distinguishable clusters.

When the differences in miRNA expression between the stimulated animals and time-matched controls were evaluated, statistically significant differences were found for the 7 d and 30 d time points (Tables 2 and 3). At 7 d after stimulation, 3 miRNAs were upregulated and 20 miRNAs were downregulated (Table 2). The most upregulated microRNAs were miR-132-3p, miR-212-3p and miR-21-5p, which were increased by 1.75, 1.83 and 1.89-fold respectively. The largest decrease in expression was observed in miR-7a-5p, miR-551b-3p and miR-187-3p at 0.53, 0.56 and 0.56-fold of the control, respectively. At the 30 d time point, changes in the expression of 20 miRNAs were observed. In comparison to the 7 d time point, a greater number of miRNAs were upregulated [11] than downregulated [9]. The most upregulated miRNAs were miR-146a-5p, miR-132-5p, miR-21-5p at 1.63, 1.61, and 1.56-fold of the control, respectively, while the most downregulated miRNAs were miR-29b-3p, miR-352, miR-30e-5p at 0.60, 0.70, and 0.72-fold of the control, respectively.

**Table 2:** miRNAs changing expression levels at 7 d after status epilepticus (FDR<0.05).

**EXIQON probe**	**miRNA**	**logFC**	**FDR**
147506	miR-21-5p*	0.919	0.011
42627	miR-212-3p	0.875	0.005
10937	miR-132-3p	0.803	0.011
148300	miR-370-5p*	0.59	0.035
148614	miR-7a-2-3p	-0.452	0.037
145633	let-7d-3p	-0.488	0.046
169394	miR-1843-5p	-0.519	0.038
148477	miR-1843-5p	-0.522	0.037
14328	miR-124-3p	-0.567	0.037
13143	miR-301a-3p	-0.569	0.049
42477	miR-324-5p	-0.601	0.037
145638	miR-29a-5p	-0.641	0.037
29190	miR-708-5p	-0.66	0.032
145742	miR-935	-0.675	0.037
145897	miR-92b-3p	-0.724	0.037
148098	miR-374-5p*	-0.727	0.017
145640	miR-328a-3p	-0.756	0.037
145677	miR-139-5p	-0.77	0.024
19596	miR-30d-5p	-0.778	0.011
145859	miR-33-5p*	-0.801	0.032
145637	miR-187-3p*	-0.842	0.032
148644	miR-551b-3p	-0.847	0.027
29490	miR-7a-5p	-0.928	0.007

* common with 30-d time point miRNAs are ordered according to decreasing logFC

**Table 3:** miRNAs that changed expression levels at 30 d after status epilepticus (FDR<0.05).

**EXIQON probe**	**miRNA**	**logFC**	**FDR**
10952	miR-146a-5p	0.702	0.032
145634	miR-132-5p	0.687	0.01
147506	miR-21-5p*	0.64	0.005
148304	miR-212-5p	0.615	0.006
42744	miR-23a-3p	0.517	0.02
168586	miR-34a-5p	0.502	0.009
148300	miR-370-5p*	0.395	0.018
29153	miR-34b-5p	0.371	0.02
42950	miR-24-2-5p	0.319	0.02
168831	miR-433-3p	0.301	0.035
11074	miR-34c-5p	0.276	0.032
14300	miR-29c-5p	-0.284	0.02
146086	miR-30a-5p	-0.3	0.02
148098	miR-374-5p*	-0.301	0.032
145859	miR-33-5p*	-0.383	0.006
11018	miR-218a-5p	-0.4	0.005
145637	miR-187-3p*	-0.463	0.033
28191	miR-30e-5p	-0.481	0.007
11273	miR-352	-0.512	0.02
11040	miR-29b-3p	-0.736	0.02

* common with 7-d time point miRNAs are ordered according to decreasing logFC

**Figure 2:**
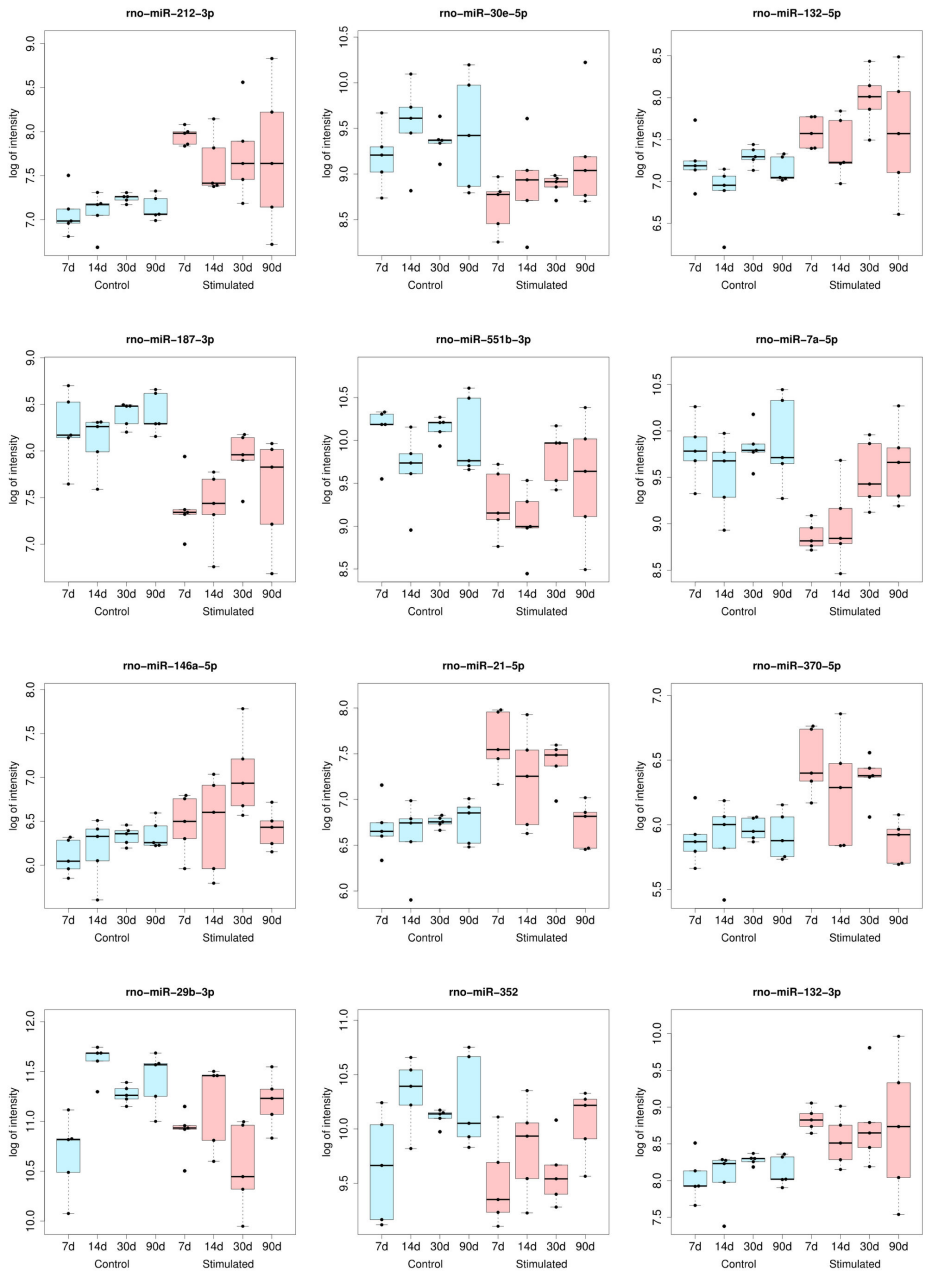
Expression levels of selected miRNAs in individual sham-operated and stimulated animals. The bottom and the top of the box indicate the first and the third quartile, the band within the box indicates the median and the ends of the whiskers represent the lowest and the highest datum still within 1.5 IQR (interquartile range) of the lower and upper quartile, respectively. Each dot represents one animal. Blue are sham operated animals, and red are stimulated animals.

We did not detect statistically significant differences (FDR<0.05) in miRNA expression between the control and stimulated animals at 14 d and 90 d. However, a number of the mean expression levels were similar to the expression levels at 7 d or 30 d after stimulation, but larger individual variability within the groups was observed (Figure 2). However, when the cut-off criteria for the selection of differentially expressed miRNA were relaxed, we could observe some changes at 14 d and 90 d after stimulation. At 14 d after stimulation, there were 4 miRNAs with different expression levels between the time-matched sham-operated and stimulated rats at unadjusted p<0.01 and 26 miRNAs at unadjusted p<0.05. At the 90 d time point, there were 2 miRNAs with different expression levels at unadjusted p<0.01 and 16 miRNAs with different expression levels at unadjusted p<0.05. We suppose that this lack of significance is due to individual differences between animals within the group (see also Figure 2). For this manuscript, we limited our analysis to the more restrictive cut-off.

**Figure 3:**
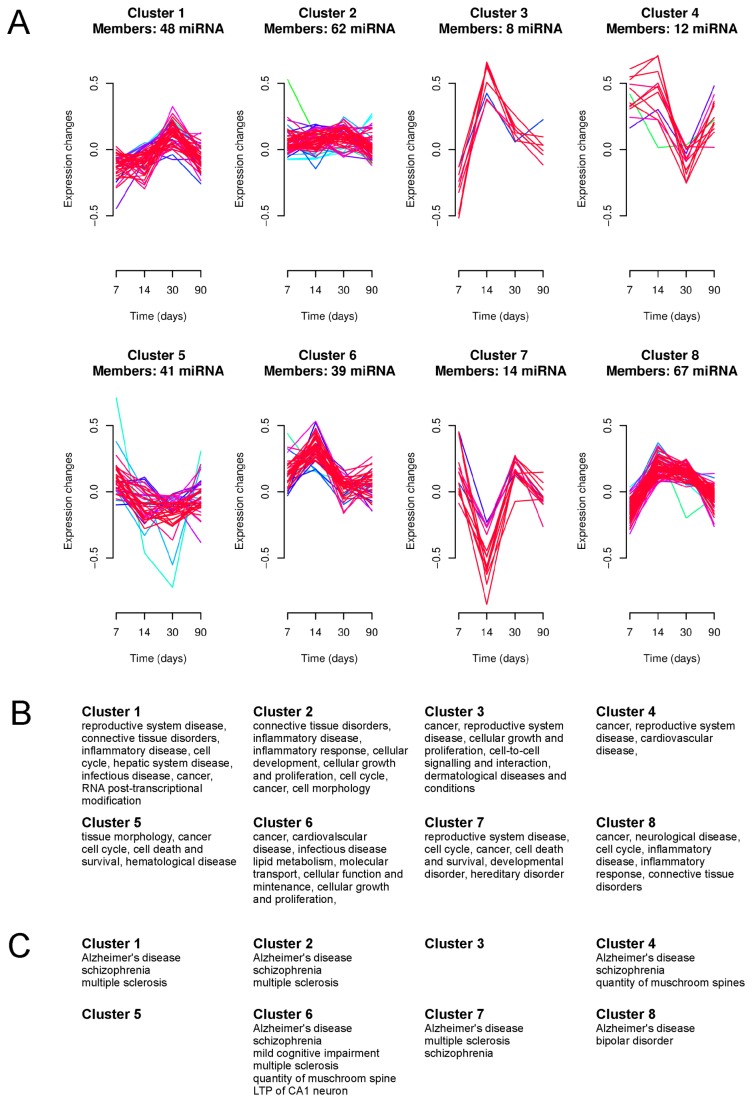
Clustering analysis of the miRNA with altered expression levels in epileptic animals. (**A**) Clusters represent groups of miRNAs displaying similar alterations in expression over time after stimulation. Colors of lines within the clusters indicate the membership values of the expression profile to current cluster. Red and violet are high membership values and blue and green are low membership values. Members of each cluster are listed in Table S1. (**B**) Functional analysis of miRNA belonging to individual clusters. Functions associated with top networks according to Ingenuity Pathways are listed. (**C**) Functions belonging to “Neurological Disorder” and “Nervous System Development and Function” categories as defined by Ingenuity Pathways in individual miRNA clusters. Functions related to brain cancer and tumors are not included. Lists of functions with respective miRNAs are presented in Table S2.

The clusterization of all 292 miRNAs that had intensities greater than the background revealed several patterns of alteration in miRNA expression over time in SE rats (Figure 3A). For example there were miRNAs regulated in opposite directions at the 7 d and 14 d time points (Figure 3A, cluster 3 and 7). Other miRNAs were upregulated only up to 14-d after SE (Figure 3A, cluster 4). We used the Ingenuity Pathway software to approach the question of the functional significance of these changes in miRNA expression (Figure 3B, C). miRNA belonging to individual clusters may be involved in several diseases. In particular, functions related to cancer are frequently represented. In several clusters, miRNA involved in neurological diseases and normal brain functions are present. miRNAs belonging to each cluster and functional classes are listed in Table S2.

**Figure 4:**
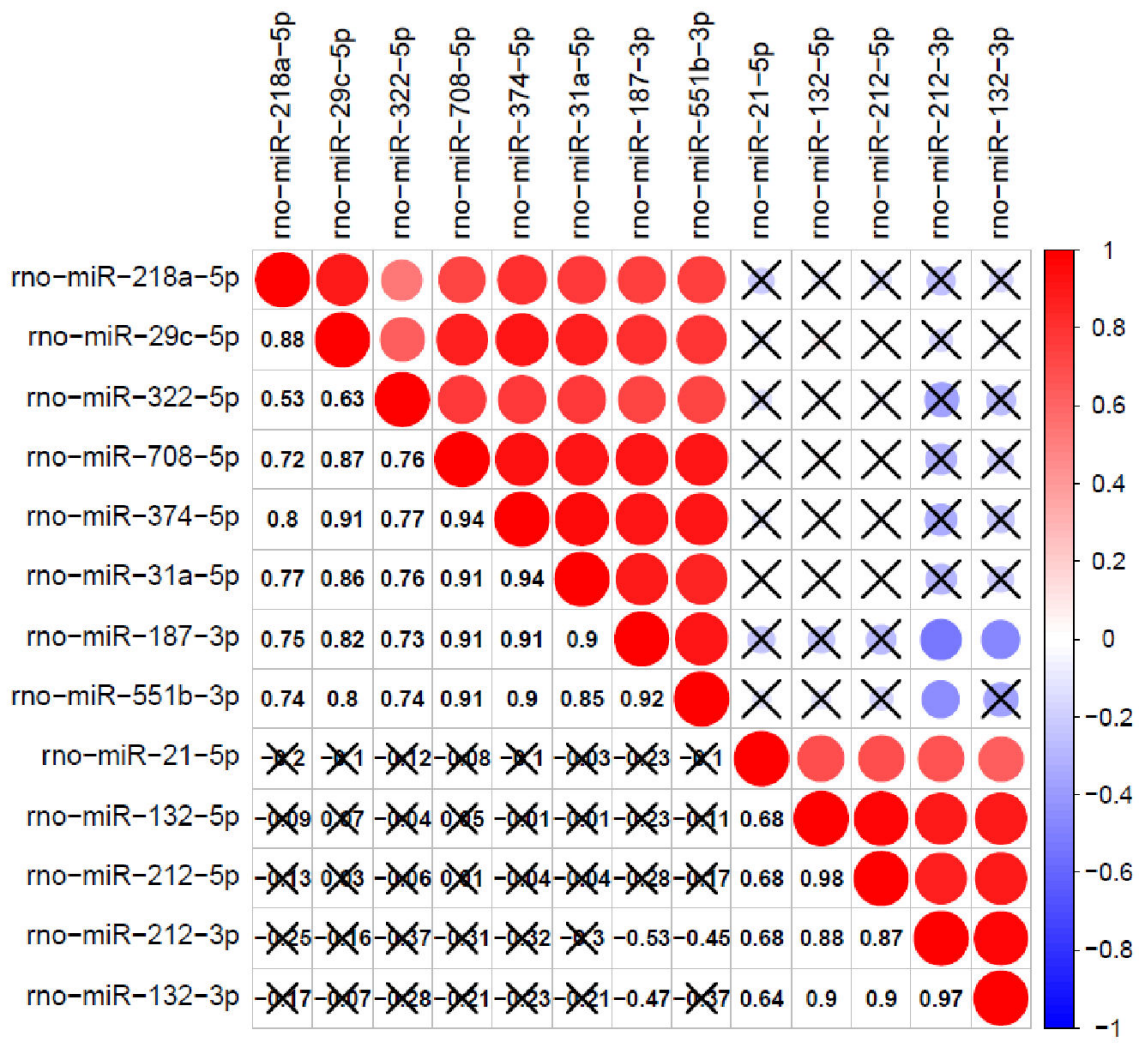
Correlation matrix of expression levels between miRNAs with expression that differs significantly between sham and stimulated animals (Table 1.) The color and size of the circles in the matrix code for level of correlation; red represents positive correlation and blue represents negative correlation. Numerical values of correlations are presented in the lower left part of the matrix. Values that do not reach statistical significance are crossed.

Interestingly, we detected correlations in the expression levels of several miRNAs. For this analysis, we included miRNAs with expression levels that were significantly different between all sham and all stimulated animals (Figure 4). The highest positive correlation of expression was observed for miR-132-5p and miR-212-5p (0.98) miR-132-3p and miR-212-3p (0.97), miR-708-5p and miR-374-5p (0.94), and miR-374-5p and miR-31a-5p (0.94).

To evaluate the potential functional impact of alterations in miRNAs expression levels, we decided to investigate if potential mRNA targets of the miRNA that change expression levels at 7 d and 30 d following SE also change their expression level. We concentrated on the expression of experimentally evaluated mRNAs defined by Ingenuity Pathways. We compared changes in miRNA expression evaluated with Exiqon microarrays and mRNA expression evaluated with Affymetrix microarrays in the dentate gyri of the same group of animals. Table 4 summarizes the potential target mRNAs that change expression in the opposite direction of the miRNA. Interestingly, several potential gene targets can be targeted by more than one miRNA. Moreover, some of these genes have been previously implicated in epileptogenesis or epilepsy (Table 4). Thus, we performed functional analysis of potential mRNA targets which revealed that target genes can be involved in several biological functions that have been previously associated with epilepsy, including regulation of transcription, response to wounding, apoptosis, cell proliferation, and immune response (Table 5).

**Table 4:** Predicted target genes for miRNAs that changed expression level at 7 d and 30 d after stimulation.

**ID**	**miRNA regulation**	**Target gene**	**p-value**	**Log Ratio**
**7 d**				
miR-212-3p	upregulated	TLN2, talin 2	0.028	-0.173
miR-21-5p	upregulated	SESN1, sestrin 1	0.033	-0.175
		BTG2, BTG family, member 2	0.004	-0.269
		IL6R, interleukin 6 receptor	0.012	-0.276
		SLC16A10, solute carrier family 16, member 10 (aromatic amino acid transporter)	0.002	-0.357
miR-124-3p	downregulated	ARPC1B, actin related protein 2/3 complex, subunit 1B	0.001	0.704
		TMBIM1, transmembrane BAX inhibitor motif containing 1	0.000	0.641
		GSN, gelsolin	0.032	0.566
		NFATC1, nuclear factor of activated T-cells, cytoplasmic, calcineurin-dependent 1	0.003	0.534
		PLP2, proteolipid protein 2	0.008	0.508
		CYP1B1, cytochrome P450, family 1, subfamily B, polypeptide 1	0.009	0.485
		CDK6, cyclin-dependent kinase 6	0.000	0.437
		RBMS1, RNA binding motif, single stranded interacting protein 1	0.018	0.434
		ELK3, ETS-domain protein (SRF accessory protein 2)	0.003	0.422
		RHOG, ras homolog family member G	0.035	0.419
		CTGF, connective tissue growth factor	0.011	0.369
		BDNF, brain-derived neurotrophic factor	0.001	0.349
		TJP2, tight junction protein 2	0.003	0.345
		TLN1, talin 1	0.009	0.344
		B4GALT1, UDP-Gal:betaGlcNAc beta 1,4- galactosyltransferase, polypeptide 1	0.002	0.321
		PTTG1IP, pituitary tumor-transforming 1 interacting protein	0.027	0.307
		MAGT1, magnesium transporter 1	0.019	0.306
		CAV1, caveolin 1, caveolae protein	0.028	0.280
		RASSF5, Ras association (RalGDS/AF-6) domain family member 5	0.006	0.273
		FAM129B, family with sequence similarity 129, member B	0.036	0.271
		RBM47, RNA binding motif protein 47	0.012	0.256
		LMNB1, lamin B1	0.011	0.253
		PLSCR3, phospholipid scramblase 3	0.045	0.240
		PTBP1, polypyrimidine tract binding protein 1	0.022	0.236
		CDK4, cyclin-dependent kinase 4	0.009	0.208
		***ATP6V0E1, ATPase, H+ transporting, lysosomal 9kDa, V0 subunit e1***	0.015	0.200
		RELA, v-rel reticuloendotheliosis viral oncogene homolog A	0.038	0.172
		DNM2, dynamin 2	0.047	0.161
		MAD2L2, MAD2 mitotic arrest deficient-like 2	0.050	0.151
		ELF4, E74-like factor 4 (ets domain transcription factor)	0.005	0.130
		ALDH9A1, aldehyde dehydrogenase 9 family, member A1	0.042	0.127
miR-301a-3p	downregulated	MAFB, v-maf musculoaponeurotic fibrosarcoma oncogene homolog B	0.001	0.576
		CSF1, colony stimulating factor 1	0.037	0.252
miR-30d-5p	downregulated	RBMS1, RNA binding motif, single stranded interacting protein 1	0.018	0.434
		CBFB, core-binding factor, beta subunit	0.044	0.403
		CTGF, connective tissue growth factor	0.011	0.369
		MET, *met* proto-oncogene (hepatocyte growth factor receptor)	0.003	0.283
		TP53, tumor protein p53	0.046	0.216
		RUNX2, runt-related transcription factor 2	0.014	0.207
		SLC4A7, solute carrier family 4, sodium bicarbonate cotransporter, member 7	0.043	0.189
miR-328a-3p	downregulated	CD44, *CD44* molecule	0.001	0.713
miR-33-5p	downregulated	ABCA1, ATP-binding cassette, sub-family A (ABC1), member 1	0.003	0.634
miR-92b-3p	downregulated	ENPP6, ectonucleotide pyrophosphatase/phosphodiesterase 6	0.003	0.846
		IKZF1, IKAROS family zinc finger 1	0.000	0.521
		CDKN1A, cyclin-dependent kinase inhibitor 1A	0.013	0.331
		ITGA5, integrin, Ralpha 5	0.033	0.170
**30 d**				
miR-146a-5p	upregulated	NFIX, nuclear factor I/X (CCAAT-binding transcription factor)	0.033	-0.090
		PA2G4, Proliferation-Associated Protein 2G	0.030	-0.113
		METTL7A, methyltransferase like 7A	0.014	-0.166
		IL1RAP, interleukin 1 receptor accessory protein	0.035	-0.170
miR-21-5p	upregulated	CDC25A, cell division cycle 25A	0.011	-0.137
		SOCS5, suppressor of cytokine signaling 5	0.007	-0.300
		IL6R, interleukin 6 receptor	0.018	-0.318
		SLC16A10, solute carrier family 16, member 10 (aromatic amino acid transporter)	0.007	-0.354
miR-23a-3p	upregulated	SMAD5, SMAD family member 5	0.013	-0.120
		IL6R, interleukin 6 receptor	0.018	-0.318
miR-34a-5p	upregulated	DLL1, delta-like 1	0.014	-0.188
		VEGFA, vascular endothelial growth factor A	0.039	-0.200
miR-29b-3p	downregulated	ZFP36L1, ZFP36 ring finger protein-like 1	0.028	0.287
		SPARC, secreted protein, acidic, cysteine-rich (osteonectin)	0.012	0.260
		COL1A2, collagen, type I, alpha 2	0.032	0.186
miR-30e-5p	downregulated	CPNE8, copine VIII	0.028	0.372
		ADPGK, ADP-dependent glucokinase	0.005	0.238
		SLC7A11, solute carrier family 7 (anionic amino acid transporter light chain, xc- system), member 11	0.041	0.230
		TP53, tumor protein p53	0.045	0.182
		TMED10, transmembrane emp24-like trafficking protein 10 (yeast)	0.020	0.177
		MPDU1, mannose-P-dolichol utilization defect 1	0.044	0.144

**Table 5:** Functional classification of potential target genes for miRNAs that changed expression levels at 7 d or 30 d after stimulation.

**Biological function**	**Gene name**
**7d**	
regulation of transcription	BTG2, IKZF1, ELF4, MAFB, RELA, TP53, ELK3, RUNX2, CBFB, NFATC1, DNM2, PA2G4, VEGFA, SMAD5, NFIX
cell motion	B4GALT1, ARPC1B, TLN1, BDNF, CD44, ITGA5, CTGF, MET, IL6R
response to wounding	B4GALT1, CD44, ITGA5, CTGF, GSN, RELA, IL6R, ELK3
regulation of cell proliferation	B4GALT1, CAV1, CSF1, RELA, TP53, CDK6, IL6R, CDK4, SESN1, BDNF, CDKN1A, BTG2, RUNX2, RHOG, PA2G4, VEGFA, TP53, CDC25A
cytoskeleton organization	ARPC1B, TLN1, CAV1, TLN2, GSN
regulation of phosphorylation	CDKN1A, CAV1, CSF1, MET, IL6R
membrane organization	CAV1, PLSCR3, TP53, ABCA1, DNM2
cation homeostasis	CAV1, ATP6V0E1, TP53
intracellular signaling	CAV1, RASSF5, CTGF, GSN, MET, TP53, ABCA1, RHOG, NFATC1, VEGFA, SMAD5, COL1A2, SOCS5
ion transport	PLP2, CAV1, ATP6V0E1, SLC4A7, NFATC1
protein localization	TLN1, CAV1, TLN2, PTTG1IP, TP53, ABCA1
regulation of apoptosis	RASSF5, GSN, TP53
immune response	IL1RAP, VEGFA, TP53, IL6R
**30 d**	
intracellular signaling	VEGFA, SMAD5, COL1A2, SOCS5, DLL1
cell proliferation	PA2G4, VEGFA, TP53, CDC25A
regulation of transcription	PA2G4, VEGFA, SMAD5, TP53, NFIX
Immune response	IL1RAP, VEGFA, TP53, IL6R
regulation of apoptosis	VEGFA, TP53, IL6R

For gene name abbreviations see Table 4.

## Discussion

Here, we show for the first time that prolonged changes in the expression of miRNAs in the rat dentate gyrus follow status epilepticus. We present lists of miRNAs that change expression levels as well as patterns of miRNA expression at different time points after status epilepticus. We also present the potential mRNA targets that could be regulated by miRNAs in the dentate gyrus and discuss the role of changes in miRNA expression in epilepsy.

miRNA has been recently implicated in a number of neurological diseases and psychiatric disorders, including brain tumors, ischemia, Alzheimer’s disease, schizophrenia, bipolar disorder and anxiety [15,42-45]. Alterations in the expression of some miRNAs have been shown in both epilepsy patients and experimental models (recently reviewed in [12]. In human temporal lobe epilepsy with hippocampal sclerosis, dysfunction of the miRNA processing machinery has been reported [21]. In the hippocampus of patients with epilepsy, McKierman et al. observed decreases in the expression of the Dicer protein, which was associated with reduced levels of mature miRNAs [21]. Kan et al. performed profiling of miRNA expression in the hippocampus and found specific miRNA expression signatures in control subjects and in epilepsy patients with and without hippocampal sclerosis [22]. A study by Abou-Zeid et al. concentrated on the role of miR-155. They showed an upregulation of miR-155 in the hippocampi of children with mesial temporal lobe epilepsy [20].

Few reports describing acute global changes in expression of miRNA following epileptogenic stimuli in experimental models have been recently published. Alterations in the expression of several miRNAs were observed in the rat hippocampus 24 hours following kainic acid-induced status epilepticus, ischemic stroke and intracerebral hemorrhage [46]. Alterations in miRNA expression were also found in hippocampal CA3 24 h following an intra-amygdala injection of kainic acid, in the hippocampus 24 h and 7 d following controlled cortical impact in rat, and in the hippocampus and hippocampal synaptoneurosomes at 4 h and 48 h following pilocarpine-induced status epilepticus [25,47,48].

Few data sets describe changes in the expression levels of miRNAs during chronic epilepsy in rats. The lithium-pilocarpine model of temporal lobe epilepsy was used in these studies. Hu et al. found an upregulation of 9 and a downregulation of 15 miRNAs in rat hippocampi 2 months after pilocarpine-induces status epilepticus [23] while Song et al. found downregulation of 5 and an upregulation of 18 miRNAs 60 d after pilocarpine application [24]. Moreover, changes in miRNA expression were studied in the hippocampus and hippocampal synaptoneurosomes at 4 h, 48 h and 3 wks following pilocarpine-induced status epilepticus [25].

In contrast to previous reports, which showed alterations in miRNA expression in the whole hippocampus, we were interested in miRNA expression profiles only in the dentate gyrus. By comparing the miRNA expression levels between all sham-operated and all stimulated animals regardless of the time point of tissue collection, we have identified a set of miRNAs that can have long-lasting effects on gene expression in the epileptic dentate gyrus (see Figure 1B, Table 1). On the other hand, the analysis of time-point specific alteration in miRNA expression between stimulated and time-matched sham-operated animals allowed us to distinguish miRNA whose actions may be more restricted in time (Figure 2, 3; Tables 2, 3). We can conclude that the patterns of miRNA expression are dynamic in time and unique for ensembles of miRNAs (Figure 3).

We have approached the issue of the role of alterations in the expression of miRNAs on the function of the epileptic brain by analyzing molecular networks that can be affected by the miRNAs detected in our study (Figure 3B, C). The Ingenuity Pathway analysis used for this analysis contains experimentally validated data on mRNAs targeted by miRNA combined with assembly of metabolic pathways also build on the basis of experimentally confirmed knowledge. Given that miRNAs have been extensively studied in cancer [49], it is not surprising that many functions assigned to miRNA clusters have been shown to be related to cancer. These include cancer, cell growth and proliferation, cell cycle, and hematological diseases. Some other functions, such as inflammatory disease and inflammatory response have been related to the pathophysiology of epilepsy because inflammation and immune response play an important role in this disease [8,50,51]. Interestingly, despite the obvious bias towards cancer related functions, we were able to detect functions specific for the brain. In particular, miRNAs from several of identified clusters are involved in Alzheimer’s disease, schizophrenia, and multiple sclerosis. Moreover, miRNA belonging to clusters 4 and 6, which increased in expression at early time points following stimulation, have a role in neuronal plasticity, because “LTP of CA1 neurons” and “quantity of mushroom spines” were assigned to these clusters. Both LTP and structural changes in spines have been previously implicated in epilepsy [52].

The other approach for understanding the functional impact of changes in miRNA expression in our experimental model was to distinguish the potential mRNA targets for miRNAs that changed expression levels in the dente gyrus. We took advantage of the transcriptome profiling data obtained from our rats and selected mRNAs that have been previously shown to be regulated by these miRNAs and changed expression levels in the dentate gyrus in the expected direction. We detected several target mRNAs that are potentially regulated by miRNA in the epileptic dentate gyrus. Protein products of these mRNAs are involved in several molecular events that occur in epileptic tissue, including the regulation of transcription, second messenger signaling, ion homeostasis, immune response, response to wounding, and regulation of cell death [8]. Interestingly, one of the potential target for several miRNAs is the receptor for interleukin 6 (IL6R). The role of cytokines, including the IL6 system has been extensively studied in epilepsy [53]. The upregulation of both IL6 and IL6R occurs following status epilepticus [54,55]. However there are no experimental data on the involvement of miRNA in the regulation of IL6R expression in the brain.

In general, the functions of the miRNAs that changed expression levels in the present experiment are related to inflammation, neuronal plasticity and neuronal development. However, the functions of even experimentally confirmed mRNA targets in the brain for the majority of miRNAs detected in our experiments, as well as for the presumed mRNA targets are not known. Altogether, understanding the impact of such orchestrated changes in miRNA expression on the function of brain tissue will require in-depth knowledge on the targets of each miRNA. At present, this knowledge is still fragmented, and for some miRNAs, it is non-existent.

Interestingly, we observed that there is an overlap between previously reported datasets derived from the epileptic hippocampus by others and our data set from the dentate gyrus. We found common expression of 9 miRNAs (miR-132, miR-137, miR-139, miR-29a, miR-324, miR-352, miR-282, miR-146a, and miR-23a) when our data were compared to a data set describing miRNA expression 60 d after pilocarpine-induced status epilepticus [24]. There are 6 common miRNAs (miR-138, miR-301a, miR-33, miR-34a, miR-146a, and miR-23a) between our data set and work of Hu et al. who studied miRNA expression profiles in a pilocarpine-induced model of epilepsy [23]. Additionally, four miRNAs (miR-138, miR-146b, miR-301a, and miR-92b) were common between our data and those of Kan et al. in human temporal lobe epilepsy [22]. Changes in the expression of miR-21 and miR-34a were found in our study and in a study by Risbud and Porter using the hippocampus from epileptic rats [25]. It can be argued that these changes in miRNAs expression detected in different brain areas, in human tissue, and in different experimental models of epilepsy are crucial for understanding the pathophysiology of the disease and may constitute interesting candidate targets for therapy. Interestingly, the role for some of these miRNAs frequently associated with epilepsy in different experimental setups has been already proposed.

For example, miR-146a is restricted to astrocytes and is overexpressed in activated astrocytes in human temporal lobe epilepsy and in experimental models [56-58]. This miRNA is crucial for regulation of astrocyte mediated inflammatory response by influencing IL-1β, IL-6 and COX-2 signaling [56].

Another miRNA, miR-132, is enriched in neurons and consistently upregulated following epileptogenic stimuli [57]. Its expression can be induced by neuronal activity and is regulated by CREB, transcription factor, which is also implicated in temporal lobe epilepsy [59]. miR-132 strongly influences neuronal morphology, increasing dendritic outgrowth and arborization [60]. It is also necessary for neuronal spine formation, and the overexpression of miR-132 results in increased spine density [61,62]. Furthermore, the overexpression or knock-down of miR-132 in the brain modulates learning and memory in several experimental paradigms [62-65]. In addition to its role in neuronal plasticity, miR-132 can participate also in neurodegeneration. In particular, following an intra-amygldala injection of kainic acid, the depletion of miR-132 reduces seizure-induced neurodegeneration [48].

Another miRNA that may be involved in neuroprotection is miR-34a. Its expression is acutely upregulated following status epilepticus and is also observed in epileptic animals [23,25]. The use of antagomirs against miR-34a decreased apoptotic markers in the hippocampus following pilocarpine-induced status epilepticus but was not effective in the intra-amygdala kainic acid injection model [23,66].

Although the effect of changes in expression of whole sets of miRNAs on function of brain tissue cannot currently be predicted there is a role for several individual miRNAs in epilepsy.

We conclude that complex changes in the expression of miRNAs in epileptic dentate gyrus and changes in expression of their potential mRNA targets suggest an important role for miRNA in molecular mechanisms of epilepsy, especially those related to inflammation and neuronal plasticity.

## Supporting Information

Table S1 **Members of gene clusters presented on Figure 3, representing groups of miRNAs displaying similar alterations in expression over time after stimulation.**
(XLS)

Table S2 **Lists of functions assigned to individual miRNAs present in clusters on Figure 3.**
(XLS)
